# Youth Use of e-Cigarette Flavor and Device Combinations and Brands Before vs After FDA Enforcement

**DOI:** 10.1001/jamanetworkopen.2023.28805

**Published:** 2023-08-14

**Authors:** Karin A. Kasza, David Hammond, Jessica L. Reid, Cheryl Rivard, Andrew Hyland

**Affiliations:** 1 Department of Health Behavior, Roswell Park Comprehensive Cancer Center, Buffalo, New York; 2 School of Public Health Sciences, University of Waterloo, Waterloo, Ontario, Canada

## Abstract

**Importance:**

The US Food and Drug Administration’s Center for Tobacco Products (CTP) prioritized its enforcement efforts against nontobacco, nonmenthol (ie, sweet)–flavored cartridge e-cigarettes in February 2020. Within-person e-cigarette initiation, continuation, and switching behaviors among youth are unknown following CTP’s prioritized enforcement efforts.

**Objective:**

To describe transitions in youths’ e-cigarette flavor/device combination use, brand use, nicotine use, and frequency of use following CTP’s e-cigarette enforcement prioritization.

**Design, Setting, and Participants:**

The US population-based, nationally representative Population Assessment of Tobacco and Health cohort study included data collected in 2019 and 2021 from youth aged 12 to 17 years. The data were analyzed from February to June 2023.

**Exposure:**

e-cigarette use (past 30 days), flavor/device combination used, brand used, nicotine use, and frequency of use.

**Main Outcomes and Measures:**

Transitions in e-cigarette use, flavor/device combination used, brand used, nicotine use, and frequency of use between 2019 and 2021 among 9088 youth aged 12 to 17 years in 2019; prevalence of e-cigarette use, flavor/device combination used, and brand used in 2019 (n = 8771) and 2021 (n = 5574) among youth aged 14 to 17 years in each year.

**Results:**

The 2019 sample included 8771 youth. The population of those aged 12 to 17 years was 49.0% female (95% CI, 48.7%-49.3%) and 51.0% male (95% CI, 50.8%-51.3%). Participants were 15.4% Black (95% CI, 15.0%-15.7%), 24.1% Hispanic (95% CI, 23.9%-24.4%), 75.9% non-Hispanic (95% CI, 75.6%-76.1%), 69.1% White (95% CI, 68.5%-69.8%), and 15.5% another race (95% CI, 14.9%-16.1%). Among youth ages 12 to 17 years who did not use e-cigarettes in 2019, 531 (6.5%) initiated use in 2021 (95% CI, 5.9%-7.1%); among them, 415 (76.8%) initiated with a combination other than a sweet cartridge (95% CI, 72.2%-80.8%). Among youth ages 12 to 17 who used e-cigarettes in 2019, 360 (47.8%) continued use in 2021 (95% CI, 44.0%-51.1%). Continuation rates were similar for those who used sweet-cartridge e-cigarettes (144 [51.5%]; 95% CI, 45.7%-57.3%) and those who used other combinations (204 [47.6%]; 95% CI, 42.8%-52.4%) in 2019. Among those who continued e-cigarette use in 2021, 121 (84.0%) of those who used sweet-cartridge e-cigarettes in 2019 switched to a different combination (95% CI, 77.0%-89.2%). Overall, among youth who used e-cigarettes in 2021, 177 (53%) used a sweet-disposable combination, 32 (11%) used a sweet-cartridge, and no individual brand was used by more than 10%.

**Conclusions and Relevance:**

The results of this longitudinal cohort study of youth in the US suggest that most youth who initiated or continued e-cigarette use in 2021 used flavor/device combinations that were excluded from CTP’s enforcement priorities. Restrictions and enforcement efforts that only cover a subset of products may be ineffective at preventing youth flavored e-cigarette use.

## Key Points

**Question:**

Did e-cigarette use among youth change following the prioritized enforcement efforts from the US Center for Tobacco Products against nontobacco, nonmenthol (ie, sweet)–flavored cartridge e-cigarettes in February 2020?

**Findings:**

This US nationally representative cohort study found no difference in e-cigarette continuation rates between youth who used sweet/cartridge e-cigarettes and youth who used other flavor/device combinations. More than 75% of youth who initiated or continued e-cigarette use in 2021 used flavor/device combinations and brands not included in the enforcement priorities of the US Center for Tobacco Products.

**Meaning:**

The results of this cohort study suggest that among youth, targeted e-cigarette enforcement was not associated with significant change in flavored e-cigarette use.

## Teaser

This cohort study examines transitions in youth e-cigarette flavor/device combination use, brand use, nicotine use, and frequency of use following e-cigarette enforcement prioritization by the US Food and Drug Administration.

## Introduction

In the US in 2019, more than 5 million youth were using e-cigarettes, including nearly 1 million using e-cigarettes every day.^1^ In January 2020, in an effort to reduce e-cigarette use among youth, the US Food and Drug Administration (FDA) Center for Tobacco Products (CTP) published guidance and enforcement priorities^2^ that prioritized enforcement efforts against “any flavored, cartridge-based ENDS [electronic nicotine delivery system] product (other than a tobacco- or menthol-flavored ENDS product).” Disposable and tank e-cigarette products were excluded from this initial enforcement guidance. However, in July 2020, CTP issued warning letters to several disposable e-cigarette brands, calling on them to remove their youth-appealing e-liquid products from the market.^3^

Cross-sectional data from the National Youth Tobacco Survey ^4,5^ and the US arm of the International Tobacco Control Policy Evaluation Project Youth Tobacco and Vaping Survey^6^ reported prevalence snapshots of e-cigarette flavors, device types, and brands used among youth before and after CTP’s 2020 enforcement guidance. These studies found that fruit-flavored and sweet-flavored e-cigarettes and a specific disposable e-cigarette brand (Puff Bar) were most popular in 2020, from which it was inferred that youth switched from fruit-flavored and sweet-flavored cartridge products to fruit-flavored and sweet-flavored disposable products.

However, to our knowledge, prior research has not reported on the distribution of e-cigarette flavors and device combinations used by US youth, which is needed to understand the potential for and effect of CTP’s 2020 enforcement guidance*.* Further, when considering leading e-cigarette brands, it is important to also consider the relative size of catch-all categories, such as “brand used is not listed” and “I do not know the name of brand used,” as there are numerous e-cigarette brands on the market.^7^ Tank/mod-style e-cigarettes are less likely to be branded than disposable or cartridge products, and such catch-all categories tend to have high rates of endorsement among youth.^4,5,6^

Further, longitudinal data are needed to identify within-person product switching, quantify e-cigarette initiation and continuation rates, and determine whether e-cigarette continuation rates were higher for those who used the products that were prioritized for CTP’s enforcement efforts than those who used products that were not, which would be expected if the enforcement efforts were effective. However, if e-cigarette continuation rates after the enforcement efforts did not differ based on the product flavor/device type used before the enforcement efforts, this would suggest that enforcement efforts were not associated with decreasing e-cigarette continuation rates as intended.

To provide a full picture of e-cigarette use among youth in the US following CTP’s enforcement guidance, we analyzed data from what is to our knowledge the first and only nationally representative longitudinal study in the US of tobacco use among youth, the Population Assessment of Tobacco and Health (PATH) Study. We report on longitudinal rates of initiation and continuation of e-cigarette flavor/device combination use, brand use, nicotine use, and frequency of e-cigarette use between 2019 and 2021 among those who were aged 12 to 17 years in 2019 and 20 years or younger in 2021. We also report on prevalence of e-cigarette use, specific e-cigarette flavor/device combinations used, and e-cigarette brands used in 2019 and 2021 among those aged 14 to 17 years and e-cigarette brands used in 2019 and 2021 among those aged 14 to 17 years in each year.

## Methods

We analyzed data from the PATH Study collected from December 2018 to November 2019 (wave 5, hereafter referred to as 2019)^8,9^ and March 2021 to November 2021 (wave 6, hereafter referred to as 2021).^9^ Data were collected using in-person and telephone audio computer-assisted self-interviews. The PATH Study was conducted by Westat and approved by the Westat institutional review board; the study examined in this article was approved by the Roswell Park institutional review board. All those aged 12 to 17 years provided assent, and their parents or legal guardians provided written informed consent. All those 18 years or older provided written informed consent. The 2019 data collection was conducted among those 12 years or older, and the 2021 data collection was conducted among those 14 years or older.^10^

We analyzed data from 3 analytic samples: 8771 youth aged 14 to 17 years in 2019 (to be consistent with the youth ages available in 2021), 5574 youth aged 14 to 17 years in 2021, and a longitudinal sample comprising 9088 youth aged 12 to 17 years in 2019 who also participated in 2021 when they were up to age 20 years. The response rate for youths in 2019 and 2021 was 84% and 64%, respectively; the response rate for adults (≥18 years) in 2021 was 74%. Nonresponse bias analysis reports were published for each wave of the PATH Study and showed that weighting adjustments essentially eliminate any subgroup overrepresentation and underrepresentation due to attrition.^10^ Further details on the PATH Study design and methods^11,12,13^ and demographic composition of the study population^14^ have been published elsewhere. Details on interviewing procedures, questionnaires, sampling, weighting, response rates, and accessing the data were previously published.^10^ This article followed the Strengthening the Reporting of Observational Studies in Epidemiology (STROBE) reporting guideline for cohort studies.

### Measures

In 2019 and in 2021, participants were asked whether they used e-cigarettes during the past 30 days, and those who had were asked which flavor they used in the past 30 days. Response options were tobacco; menthol or mint (asked as 1 category in 2019 and 2 categories in 2021); clove or spice; fruit; chocolate; an alcoholic drink; a nonalcoholic drink; candy, desserts, or other sweets; or some other flavor. We relied on which flavor participants selected to categorize their flavor. Respondents could endorse more than 1e flavor, and those who did in 2021 were asked which flavor(s) they used most often. We categorized e-cigarette flavors into 6 mutually exclusive and exhaustive categories: (1) only tobacco flavor; (2) only menthol/mint flavor; (3) only nontobacco, nonmenthol/mint flavor (hereafter referred to as sweet because nearly all flavors used were sweet), (4) sweet and menthol/mint flavor (no tobacco flavor), (5) menthol/mint and tobacco flavor (no sweet flavor), and (6) sweet and tobacco flavor (with or without menthol/mint flavor).

During each year, those who had used e-cigarettes were also asked if the product they used most often was a disposable device, device that uses replaceable prefilled cartridges, device with a tank that is refilled with liquids, mod system, or something else/not defined. We categorized e-cigarette device type into 3 mutually exclusive and exhaustive categories: (1) disposable products, (2) cartridge products, and (3) tank or mod systems. We generated a 19-level e-cigarette flavor-by-device combination variable in which we crossed the 6 flavors with the 3 device types (18 categories), and we included those who had missing data on either flavor or device type as an additional unknown category. We also collapsed the 19-level flavor/device combination variable into a 3-level variable to align specifically with CTP’s 2020 e-cigarette enforcement guidance: (1) those who used any sweet flavor in a cartridge device (ie, the previously described flavor categories 3, 4, and 6, combined with the previously described device category 2), (2) those who reported any other flavor/device category, and (3) those who had missing data on either flavor or device (ie, unknown flavor/device combination variable as described previously).

Those who had used e-cigarettes were asked whether they knew the name of the brand of e-cigarettes that they usually used, and if so, they were asked to name the brand by selecting from a list of hundreds of brands (the list updated each year) or by writing in the brand name if it was not listed (they could also select “don’t know”). Separately in 2019 and 2021, among those who used e-cigarettes, we calculated the fraction who did not know the name of the brand they usually used and the remaining fraction who named a brand, and we identified the most frequently named brands in each year. Those who used e-cigarettes were also asked whether the e-cigarettes they use most often contained nicotine (response options: yes, no, or I do not know) and their frequency of e-cigarette use (number of days they used e-cigarettes in the past 30 days).

Lastly, respondents or their legal guardians reported respondent age, biological sex (female or male), race (Black or African American, White, or another race [ie, American Indian or Alaska Native, Asian Indian, Chinese, Filipino, Guamanian or Chamorro, Japanese, Korean, Native Hawaiian, Samoan, Vietnamese, Other Asian, or Other Pacific Islander]), and ethnicity (Hispanic or not Hispanic).

### Statistical Analyses

#### Longitudinal Transitions in e-Cigarette Use Between 2019 and 2021

First, among those who had not used e-cigarettes during the past 30 days in 2019, we reported rates of initiating e-cigarette use in 2021 (ie, the transition from no past 30-day use in 2019 to past 30-day use in 2021; may have ever or never used in 2019). Among those who initiated e-cigarette use in 2021, we reported initiation rates by flavor/device combination, brand, nicotine use, and frequency of use. Second, among those who used e-cigarettes during the past 30 days in 2019, we reported rates of continuing e-cigarette use in 2021 (ie, past 30-day use in 2019 and past 30-day use in 2021) overall and stratified by 2019 flavor/device combination used, brand used, nicotine used, and frequency of e-cigarette use. Among those who used e-cigarettes in 2019 and continued e-cigarette use in 2021, we reported rates of switching or continuing flavor/device combination use. The 2019 to 2021 longitudinal analyses were weighted using the 2021 all-waves weights so that they represented the resident population of the US in 2021 who were in the civilian, noninstitutionalized population in 2016 and 2017. All variances were estimated using the balanced repeated replication method,^15^ with Fay adjustment set to 0.3 to increase estimate stability.^16^ Design-based F tests were used to compare rates. Stata, version 17 (StataCorp), was used for analyses. An α of .05 was considered statistically significant.

#### Prevalence of e-Cigarette Use and Characteristics of e-Cigarettes used in 2019 and 2021

Among those aged 14 to 17 years in 2019, and separately among those aged 14 to 17 years in 2021, we reported the prevalence of (1) past 30-day e-cigarette use, (2) e-cigarette flavor/device combination used among those who used e-cigarettes (using the 19-level e-cigarette flavor/device combination variable), and (3) e-cigarette brand use among those who used e-cigarettes. The 2019 and 2021 prevalence estimates were weighted using single-wave weights to represent the resident population of the US in 2019 and in 2021, respectively, as described elsewhere.^10^

## Results

The population of those aged 12 to 17 years in 2019 was 51.0% (95% CI, 50.8%-51.3%) male and 49.0% female (95% CI, 48.7%-49.3%). Participants were 15.4% Black (95% CI, 15.0%-15.7%), 24.1% Hispanic (95% CI, 23.9%-24.4%), 75.9% non-Hispanic (95% CI, 75.6%-76.1%), 69.1% White (95% CI, 68.5%-69.8%), and 15.5% another race (95% CI, 14.9%-16.1%).

### Transitions in e-Cigarette Use Between 2019 and 2021

#### e-Cigarette Initiation

Table 1 shows longitudinal transitions in e-cigarette use among youth aged 12 to 17 years in 2019. Overall, among those who did not use e-cigarettes in 2019, 531 (6.5%) initiated e-cigarette use in 2021 (Table 1). Among those who initiated e-cigarette use in 2021, 415 (76.8%) initiated with a flavor/device combination other than a sweet flavor in a cartridge device, and 54 (11.4%) initiated with a sweet flavor in a cartridge device (Table 1). Among those who initiated e-cigarette use in 2021, 40 (8.0%) initiated with Hyde brand e-cigarettes, 40 (6.6%) initiated with Puff Bar, 27 (5.6%) initiated with Juul, 28 (5.2%) initiated with Vuse, and the remaining 396 (74.6%) initiated with some other brand or did not know the brand (Table 1). Most (466 [86.8%]) of those who initiated e-cigarette use in 2021 used e-cigarettes that contained nicotine, and close to one-third (169 [30.6%]) of those who initiated used e-cigarettes on 20 or more days during the previous 30 days (Table 1).

**Table 1.** e-Cigarette Initiation in the US Between 2019 and 2021 Among Youth Aged 12 to 17 Years Who Did Not Use e-Cigarettes in 2019

Characteristic	e-Cigarette initiation in 2021, No. (%) [95% CI]
Among those who did not use e-cigarettes in 2019 (n = 8315)	531 (6.5) [5.9-7.1]
Among those who initiated e-cigarette use between 2019 and 2021 (n = 531)	NA
e-Cigarette flavor/device combination used in 2021	
Any sweet flavor + cartridge device^a^	54 (11.4) [8.8-14.7]
All other flavor/device combinations, known	415 (76.8) [72.2-80.8]
Unknown flavor/device combinations	62 (11.9) [8.8-15.8]
e-Cigarette brand used in 2021	
Hyde	40 (8.0) [5.6-11.5]
Puff bar	40 (6.6) [4.8-9.2]
Vuse	27 (5.2) [3.4-7.7]
Juul	28 (5.6) [3.9-8.0]
All other known/unknown	396 (74.6) [70.3-78.4]
e-Cigarette containing nicotine used in 2021	
Yes	466 (86.8) [82.8-89.9]
No	36 (6.5) [4.4-9.5]
Do not know	29 (6.8) [4.4-10.3]
Frequency of use in 2021 (No. of days used e-cigarettes in past 30 d)	
1-5 d	224 (43.0) [38.1-48.0]
6-19 d	85 (16.0) [12.9-19.7]
20-30 d	169 (30.6) [26.6-35.0]
Not reported	53 (10.4) [7.8-13.7]

Abbreviation: NA, not applicable.

^a^ Any sweet flavor included use of any of the following flavors (with or without use of tobacco flavor or menthol/mint flavor): fruit, chocolate, candy, desserts, other sweets, alcoholic drink, nonalcoholic drink, clove, spice.

#### e-Cigarette Continuation

Overall, among those who used e-cigarettes in 2019, 360 (47.8%) continued e-cigarette use in 2021 (ie, used e-cigarettes during the past 30 days in 2019 and used e-cigarettes during the past 30 days in 2021; Table 2). e-Cigarette continuation rates were similar among those who in 2019 used any sweet flavor and a cartridge device (144 [51.5%]) and those who used any other known flavor/device combination (204 [47.6%]) and were lower among those who in 2019 did not know the flavor/device combination that they used (12 [24.6%]). e-Cigarette continuation rates were similar among those who used known brands in 2019 (ranging from 57.9%-63.6%) and were lower among those who did not know the name of the e-cigarette brand used in 2019 (196 [40.5%]; Table 2). e-Cigarette continuation rates were higher among those who in 2019 used e-cigarettes with nicotine (291 [54.3%]) than those who used e-cigarettes without nicotine (43 [32.4%]). Lastly, e-cigarette continuation rates were highest among those who in 2019 used e-cigarettes on 20 or more days during the previous month (140 [71.7%]) and lowest for those who used e-cigarettes on 5 or fewer days during the previous month (130 [35.6%]; Table 2).

**Table 2.** e-Cigarette Continuation in the US Between 2019 and 2021 Among Youth Aged 12 to 17 Years Who Used e-Cigarettes in 2019

Characteristic	e-Cigarette continuation in 2021, No. (%) [95% CI]
Among those who used e-cigarettes in 2019 (n = 773)	360 (47.8) [44.0-51.1]
e-Cigarette flavor/device combination used in 2019	
Any sweet flavor + cartridge device (n = 291)^a^	144 (51.5) [45.7-57.3]
All other flavor/device combinations, known (n = 435)	204 (47.6) [42.8-52.4)
Unknown flavor/device combination (n = 47)	12 (24.6) [13.8-39.9]
e-Cigarette brand used in 2019	
Juul (n = 170)	103 (61.0) [52.4-69.0]
Blu cigs (n = 25)	14 (61.5) [40.0-79.3]
Smok (n = 18)	11 (63.6) [39.3-82.5]
Other known brands (n = 63)	36 (57.9) [46.6-68.4]
Unknown brands (n = 497)	196 (40.5) [36.4-44.8]
e-Cigarette containing nicotine used in 2019	
Yes (n = 539)	291 (54.3) [50.4-58.2]
No (n = 136)	43 (32.4) [23.4-42.9]
Do not know (n = 98)	26 (30.7) [21.8-41.3]
Frequency of use in 2019 (No. of days used e-cigarettes in past 30 d)	
1-5 d (n = 413)	130 (33.6) [28.8-38.7]
6-19 d (n = 143)	75 (53.3) [43.7-62.6]
20-30 d (n = 192)	140 (71.7) [65.0-77.5]
Not reported (n = 25)	15 (55.0) [33.8-74.6]

^a^ Any sweet flavor included use of any of the following flavors (with or without use of tobacco flavor or menthol/mint flavor): fruit, chocolate, candy, desserts, other sweets, alcoholic drink, nonalcoholic drink, clove, and spice.

#### e-Cigarette Flavor/Device Combination Switching

Among those who continued e-cigarette use in 2021, 121 (84.0%) of those who used a sweet flavor in a cartridge device in 2019 switched to using a flavor/device combination other than a sweet flavor in a cartridge device in 2021. A total of 34 (16.1%) of those who used a flavor/device combination other than a sweet flavor in a cartridge device in 2019 switched to using a sweet flavor in a cartridge device in 2021 (Table 3).

**Table 3.** e-Cigarette Flavor/Device Combination Switching or Continuation in the US Between 2019 and 2021 Among Youth Aged 12 to 17 Years Who Used e-Cigarettes in 2019 and 2021

Characteristic	2021, No. (%) [95% CI]		
	Any sweet flavor + cartridge device^a^	All other flavor/device combinations, known	Unknown flavor/device combinations
Among those who used e-cigarettes in 2019 and 2021 (n = 360)	54 (15.0) [11.3-19.6]	290 (80.3) [75.4-84.4]	16 (4.7) [2.9-7.6]
Flavor/device combination used in 2019^b^			
Any sweet flavor + cartridge device (n = 144)^a^	19 (13.6) [8.5-21.0]	121 (84.0) [77.0-89.2]	4 (2.4) [1.1-5.0]
All other flavor/device combinations, known (n = 204)	34 (16.1) [11.6-22.0]	160 (78.3) [72.1-83.4]	10 (5.6) [3.0-10.3]

^a^ Any sweet flavor included use of any of the following (with or without tobacco flavor or menthol/mint flavor): fruit, chocolate, candy, desserts, other sweets, alcoholic drink, nonalcoholic drink, clove, and spice flavors.

^b^ Excluding 12 unknown flavor/device combinations.

### Prevalence of e-Cigarette Flavor/Device Combinations Used in 2019 and 2021

Among youth aged 14 to 17 years, 1042 (12%) used e-cigarettes during the past 30 days in 2019 (ie, December 2018 to November 2019), and 325 (6%) used e-cigarettes during the past 30 days in 2021 (ie, March 2021 to November 2021). Among those who used e-cigarettes in 2019, 213 (19%) used a sweet-tank flavor/device combination, 184 (18%) used a sweet-cartridge combination, 172 (17%) used a sweet plus menthol/mint–cartridge combination, and less than 15% used each of the other 16 flavor/device combinations (Figure, A). Among those who used e-cigarettes in 2021, 105 (31%) used a sweet-disposable flavor/device combination, 66 (20%) used a sweet plus menthol/mint–disposable combination, and less than 11% used each of the other 17 flavor/device combinations (Figure, B).

**Figure.**
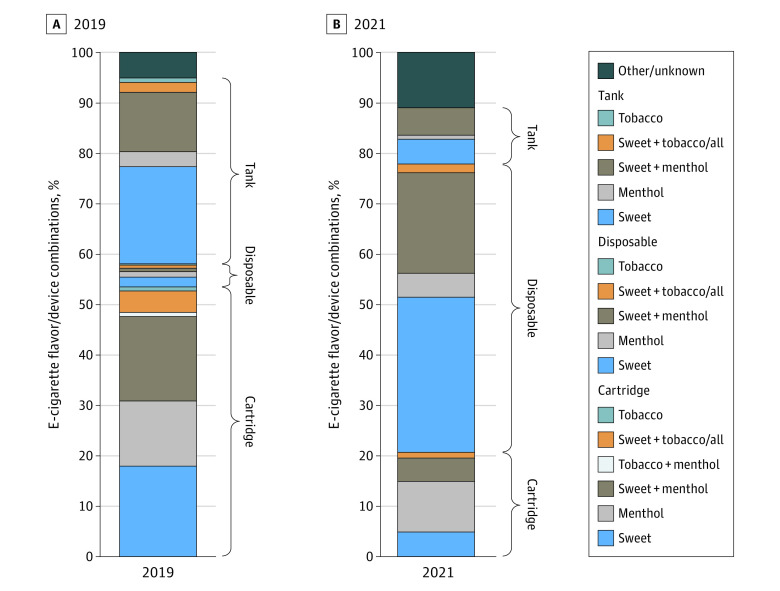
e-Cigarette Flavor/Device Combinations Used Among 1042 and 325 Youth Who Used e-Cigarettes During the Past 30 Days in 2019 and 2021, Respectively Sweet included only fruit, chocolate, candy, desserts, other sweets, alcoholic drink, nonalcoholic drink, clove, and spice flavors. Sweet+menthol included only sweet flavor and menthol/mint flavor. Menthol included only menthol/mint flavor. Sweet+tobacco/all included any sweet flavor and tobacco flavor and any sweet flavor and tobacco flavor and menthol/mint flavor. Tobacco included only tobacco flavor. Tobacco+menthol included only tobacco flavor and menthol/mint flavor. A, The prevalence of e-cigarette brands used in 2019 was 24% Juul, 3% Blu cigs, 2% Smok, 1% E-Swisher, 1% NJOY, 63% did not know brand, and the remaining 6% used various other brands. B, Device categories for tobacco and for tobacco+menthol were combined due to small sample sizes. The prevalence of e-cigarette brands used in 2021 was 10% Hyde, 9% Puff Bar, 8% Vuse, 7% Juul, 1% NJOY, 47% did not know brand, and the remaining 18% used various other brands.

## Discussion

This nationally representative longitudinal US cohort study found no differences in e-cigarette continuation rates among youth who used the flavor/device combination prioritized for CTP’s enforcement efforts in 2020 (ie, a sweet flavor in a cartridge device) compared with youth who used other e-cigarette flavor/device combinations. We found that 84% of youth who used sweet/cartridge e-cigarettes in 2019 and continued e-cigarette use in 2021 switched to using a different flavor/device combination. These findings provide what is to our knowledge the first longitudinal evidence that CTP’s partial e-cigarette enforcement efforts left open an avenue through which youth continued use of flavored e-cigarettes through using devices not covered by the enforcement guidance. The changes we observed likely also reflect volatility in the e-cigarette market more generally. For example, youth and young adult use of cartridge Juul brand e-cigarettes decreased in England and Canada where there were no device specific restrictions.^17^

This study’s longitudinal findings are reinforced by consistency in the overall e-cigarette flavor use prevalence among youth in 2019 and 2021 (Figure) alongside the relative expansion in the fraction of youth who used disposable products and contraction in the fraction who used cartridge products, findings that were consistent with cross-sectional data sources.^4,5,6^ Multiple sources identified that the disposable e-cigarette market, which contains inexpensive flavored products that appeal to youth, began proliferating in late Spring 2019.^18^ Restrictions and enforcement efforts that only cover a subset of flavored e-cigarettes do not appear to be associated with preventing youth flavored e-cigarette use. Massachusetts, Rhode Island, New Jersey, and New York have implemented comprehensive flavored e-cigarette sales restrictions,^19^ and e-cigarette sales data show declines in sales of nontobacco-flavored e-cigarettes in these states.^20^ However, population-based survey data have shown continued use of flavored e-cigarettes among youth,^21^ pointing to unrealized opportunities for compliance and enforcement efforts at state levels. Taken with the findings from this study, there appears to be unrealized potential at the national level to prevent youth access to flavored e-cigarette products.

Near to CTP’s prioritization of its e-cigarette enforcement efforts in the beginning of 2020, the federal minimum age for sale of tobacco products (including e-cigarettes) was raised from 18 years to 21 years (“Tobacco 21”) at the end of 2019,^22^ the US Centers for Disease Control and Prevention identified an outbreak of e-cigarette-associated lung injuries in August 2019,^23^ and a public health emergency was declared due to the spread of COVID-19 in January 2020.^24^ COVID-19 may have hindered CTP’s ability to execute its enforcement priorities, though the agency reported having issued warning letters to numerous companies found to be selling unauthorized products after CTP implemented its enforcement priorities.^25^

All of these co-occurring policies and events likely contributed to the decrease in e-cigarette use prevalence among youth that we observed between 2019 and 2021 and that was found with the National Youth Tobacco Survey.^26^ However, key to the present study’s findings is that we found no evidence of differences in e-cigarette continuation rates between youth who previously used e-cigarettes targeted by CTP and youth who used other e-cigarettes. There was no flavor/device type specificity in whether youth continued to use e-cigarettes after CTP’s targeted enforcement efforts, as would be expected if the efforts were effective at motivating e-cigarette cessation rather than product switching.

### Limitations

Some important unanswered questions remain following this study. For example, it is unknown whether restrictions on all sweet flavors (regardless of device type) would be associated with decreased e-cigarette continuation rates or whether sweet vs nonsweet flavored e-cigarette use among people who smoke cigarettes is associated with differences in cigarette continuation rates. Also, the PATH Study 2021 data were collected using telephone and in-person interviews, whereas the 2019 data were all collected in-person, and studies have shown there are differences in who responds by and the responses provided via telephone vs in-person interviews.^27,28,29^ In the PATH Study in 2021, in-person data collection was prioritized when permissible by Westat, the National Institute on Drug Abuse, US Food and Drug Administration, and local jurisdictions in compliance with local and state restrictions for COVID-19 mitigation, although participants who were eligible for in-person interviews could choose to be interviewed by telephone instead.^10^ The PATH Study 2021 mode of data collection is inextricably tied to time-specific and place-specific COVID-19 risk and mitigation measures; therefore, differences in estimates yielded between modes are expected. In this article, we included in the analyses respondents who participated via either mode of data collection, and we weighted our estimates to statistically represent the entire resident population aged 14 to 20 years in 2021 who were in the population during 2016 and 2017.^10^

## Conclusions

In this cohort study that examined CTP’s e-cigarette enforcement prioritization in 2020, we found no evidence of differences in e-cigarette continuation rates between youth who previously used the targeted e-cigarettes and youth who used other e-cigarettes. Most youth who used the targeted products in 2019 and continued e-cigarette use in 2021 had switched to flavor/device combinations that were excluded from CTP’s enforcement priorities. Restrictions and enforcement efforts that only cover a subset of products do not appear to be associated with preventing youth flavored e-cigarette use.

## Notes

Supplement.
Data sharing statement
