# Supplementary material for: Youth Use of e-Cigarette Flavor and Device Combinations and Brands Before vs After FDA Enforcement
Source: JAMA Netw Open. 2023 Aug 14;6(8):e2328805. doi: 10.1001/jamanetworkopen.2023.28805 (PMC10425823; doi:10.1001/jamanetworkopen.2023.28805)
Supplement: Supplement. — Data sharing statement [file jamanetwopen-e2328805-s001.pdf]

Data Sharing Statement

Kasza. Youth Use of e-Cigarette Flavor and Device Combinations and Brands Before vs After FDA Enforcement. *JAMA Netw Open*. Published August 14, 2023.
doi:10.1001/jamanetworkopen.2023.28805

Data
Data available: Yes
Data types: Deidentified participant data, Data dictionary
How to access data: Data from the Population Assessment of Tobacco and Health Study waves 1-6 may be obtained from a third party and are not publicly available (https://www.icpsr.umich.edu/icpsrweb/NAHDAP/studies/36231). Application instructions and conditions of use are available at the website mentioned.
When available: With publication

Supporting Documents
Document types: None

Additional Information
Who can access the data: Researchers whose proposed use of the data has been approved by a third party. Application instructions and conditions of use are available at https://www.icpsr.umich.edu/icpsrweb/NAHDAP/studies/36231
Types of analyses: For any purpose approved by a third party. Application instructions and conditions of use are available at https://www.icpsr.umich.edu/icpsrweb/NAHDAP/studies/36231
Mechanisms of data availability: With a signed data access agreement with a third party. Application instructions and conditions of use are available at https://www.icpsr.umich.edu/icpsrweb/NAHDAP/studies/36231
